# The Regenerative Potential of Donkey and Human Milk on the Redox-Sensitive and Proliferative Signaling Pathways of Skin Fibroblasts

**DOI:** 10.1155/2020/5618127

**Published:** 2020-11-11

**Authors:** H. Kocic, T. Langerholc, M. Kostic, S. Stojanovic, S. Najman, M. Krstic, I. Nesic, A. Godic, U. Wollina

**Affiliations:** ^1^Clinic for Dermatology, University Clinical Center Faculty of Medicine, Nis, Serbia; ^2^Faculty of Agriculture, University of Maribor, Maribor, Slovenia; ^3^Medical Faculty, University of Nis, Nis, Serbia; ^4^Department of Dermatology, University Hospital Lewisham, London SE13 6LH, UK; ^5^Department of Dermatology and Allergology, Stadtisches Klinikum Dresden, Dresden, Germany

## Abstract

The influence of milk bioactive peptides on skin regenerative potential and rejuvenation is very often limited because of allergic reactions. The current study is aimed at exploring the influence of donkey colostrum and mature milk, human colostrum and mature milk, and *β*-casein and *β*-casomorphine-7, on the growth and inflammatory response of the culture of cultured skin fibroblasts exposed to these conditions for twenty-four hours. Their effects on the growth-regulatory kinases and redox-sensitive, proinflammatory transcriptional factor NF-*κ*B were detected by using specific primary antibodies against NF-*κ*B p65, Akt-1, phospho-Akt-1, Erk-1, phospho-Erk-1, JNK, phospho-JNK, phospho-STAT-1, and CD26, while logarithmic integrated fluorescence intensity patterns were recorded by flow cytometry. The downregulation of NF-*κ*B p65 was observed after the exposure of skin fibroblasts to donkey milk and human colostrum, while *β*-casein and *β*-casomorphine-7 exerted the opposite effect, which suggests that noncasein bioactive peptides of donkey and human milk may be responsible for anti-inflammatory properties. The exposure to all milk species examined and *β*-casein leads to the activation of growth-regulatory kinases (Akt1/2/3 kinase, Erk kinase, JNK kinase, and Stat-1 kinase), especially for the p-Erk pathway, which suggests that essential amino acids of casein may be responsible for Erk-induced cell cycle activation and proliferation. The opposite effect was observed when cells were exposed to *β*-casomorphine-7, which may affect the skin fibroblast survival and their proliferative and regenerative potential. Donkey milk did not significantly change the CD26 antigen expression. In conclusion, our results suggest that among cell signaling molecules, the most sensitive but nonspecific downstream effector is p-Erk kinase, which may point to donkey milk usefulness in wound healing, regenerative, and aesthetic dermatology. The noncasein bioactive peptides of donkey milk may be responsible for the anti-inflammatory property of donkey milk and colostrum, which may indicate the usefulness in the treatment of inflammatory skin diseases.

## 1. Introduction

Skin fibroblasts exert a primary biological role in production and degradation of the extracellular matrix (ECM) [1–4]. The ECM consists of collagen, adhesive proteins, and other space-filling substances. Environmental and metabolic factors, immunological stimulators, UV irradiation, and other epigenetic factors may modulate fibroblast regulatory kinases, oxidant-sensitive transcriptional factors, or apoptotic proteins signaling, having a profound influence on growth, differentiation, apoptosis, and systemic response. Inflammatory cytokines, mainly interleukins (IL-1*β*, IL-6, IL-13, IL-33), prostaglandins, transforming factor-*β* (TGF-*β*), and leukotrienes, can induce a transition of skin fibroblasts to myofibroblasts and may upregulate fibroblasts chemotaxis, collagen production, or secretion of their inflammatory cytokines and reactive oxygen species. Their uncontrolled stimulation may be harmful, resulting in an enormous fibrotic response or chronic systemic inflammation [5–7]. The identification and characterization of the cell-signaling pathways for skin fibroblast may be valuable in developing therapeutic approaches in skin regeneration and wound healing [8–10]. Additionally, the inhibition of some surface proteins with peptidase activity, such as the antigen CD26, may reduce skin scarring [11–14].

Among the growth-regulatory kinases, the key regulators of cellular metabolism, proliferation, regeneration, and differentiation are Akt kinase and the mitogen-activated protein kinases (MAPKs), mainly Erk kinases [15–19]. A family of JNK kinases and transcription factor NF-*κ*B can respond to different oxidative stress stimuli and to cytokine stimulation. Signal transducers and activators of transcription (Stat) represent a family of proteins, which can serve as transcription factors to a variety of growth factors and cytokines. The process of wound healing proceeds into three phases: inflammation, tissue formation, and tissue remodeling [20]. On the other hand, programmed cell death or apoptosis dysregulation may induce excessive scarring and fibrosis. Different cell signals may be involved in the induction of apoptosis controlling the tissue repair. In a wound healing process, both, the production of growth factors and myofibroblast apoptosis, orchestrate the phases of tissue repair [21, 22].

Milk proteins provide an important source of bioactive peptides, which can demonstrate specific physiological functions and some of them hormone-like activities. These effects may be local or systemic [23]. So far, opiate, antithrombotic, antihypertensive, immunomodulatory, antioxidative, antimicrobial, anticancer, mineral-carrying, and growth-promoting properties have been reported. A low casein/whey protein ratio may play a role in preventing allergenic sensitization to constituent cows' milk proteins [24–29]. Low casein content may also decrease the presence and harmful effect of partially degraded bioactive casein derivatives, such as beta casomorphin-7 [23, 30, 31].

We hypothesized that the treatment of human fibroblasts with donkey milk and human milk, casein, and beta-casomorphine-7 might modulate their growth-regulatory kinases, redox-sensitive transcriptional nuclear factor kappa B (NF-*κ*B), and immunocompetent antigen CD26 expression through epigenetic mechanisms, which might have an influence on skin fibroblasts proliferative and inflammatory potential. We report the effect of different milk species on the expression of NF-*κ*B; Akt-1, 2, 3; phospho-Akt-1, 2, 3; Erk-1/2; phospho-Erk-1/2; JNK; phospho-JNK; phospho-Stat-1; and CD26 in L929 skin fibroblasts culture.

## 2. Materials and Methods

### 2.1. Milk Sample Collection

All milk samples were collected according to the stage of lactation and equilibrated to protein level. Donkey colostrum samples were collected from five donkeys (*Equus asinus asinus*) one day after the delivery. In addition, samples of mature milk were collected from the same animals three months after the delivery. Human breast milk was collected from five puerperal women who had delivered healthy infants at full-term without medical complications during pregnancies. Informed consents to participate in the study were also obtained. The first milk sample (colostrum) was collected immediately after the first appearance of milk; the second sample was collected one month afterwards. The milk samples collected were prepasteurized at 60°C for 5 min and kept at -17°C.

### 2.2. Cell Culture

Mouse skin fibroblasts (L929 fibroblast cell line) were purchased from the American Type Culture Collection (ATCC, Manassas, Virginia, USA). The cells were maintained in Dulbecco's Modified Eagle Medium (DMEM GibcoBRL, Rockville, MD, USA), supplemented with 10% fetal calf serum (FCS), 2 mM glutamine, 50 U/mL penicillin, and 50 *μ*g/mL streptomycin at 37°C in a 5% CO_2_ incubator. The cells were plated in 24-well tissue culture dishes (2 × 10^6^ cells/well), using 1 mL of medium in each well. Forty-eight hours later, the milk samples, diluted 1/100 in medium, were added to the wells and incubated at 37°C for further twenty-four hours. The concentration of *β*-caseine and *β*-casomorphine-7 was calculated according to their approximate dose in milk and diluted 1/10 (dose I) and 1/100 (dose II) [32]. The groups of fibroblasts examined were treated with the following milk/protein samples: donkey colostrum (donkey 1); donkey mature milk (donkey 2); human colostrum (human 1); human mature milk (human 2); casein 1/10, i.e., 2.4 mg/mL of medium (cas I); casein 1/100, i.e., 0.24 mg/mL of medium (cas II); beta-casomorphine-7, 1/10, i.e., 3 *μ*g/mL of medium (CSM I); beta-casomorphine-7, 1/100, i.e., 0.3 *μ*g/mL of medium (CSM II), and control group maintained in above mentioned culture conditions (DMEM supplemented with 10% FCS, 2 mM glutamine, 50 U/mL penicillin, and 50 *μ*g/mL streptomycin). Each group consisted of 4-6 culture samples.

### 2.3. Flow Cytometry

Specific primary antibodies against NF-*κ*B p65 (sc-372), Akt-1 (sc-56878), phospho-Akt-1 (sc-135651), Erk-1 (sc-135900), phospho-Erk-1 (sc-136521), JNK (sc-7345), phospho-JNK (sc-293137), phospho-STAT-1 (sc-51700), and CD26 (sc-9153) were purchased from Santa Cruz Biotechnology (Santa Cruz, CA, USA). As a staining control, a mouse IgG was used in combination with a FITC-goat-anti-mouse secondary antibody. Cells were fixed in 4% formaldehyde and permeabilized with 0.1% Triton X-100 in PBS (pH 7.4). To avoid any nonspecific binding and background fluorescence during immunostaining, the cells were incubated with specific primary antibodies at 4°C in PBS containing 1% BSA overnight. After that, they were washed and incubated at room temperature for 1 h with corresponding FITC-conjugated secondary antibodies (goat anti-mouse IgG, sc-2010, mouse anti-goat IgG, sc-2354, and goat anti-rabbit IgG, sc-2012), purchased from Santa Cruz Biotechnology (CA, USA). The cells were then analyzed for corresponding cell signaling molecules by flow cytometry on a LSR BD Fortessa Flow cytometer. The obtained logarithmic integrated fluorescence intensity patterns were recorded and analyzed by software BD FACSDiva™, version 8.0.

### 2.4. Cell Viability Assay

Skin fibroblast cell lines were seeded in 96-well plates (Greiner Bio-One, Frickenhausen Germany) at a density of 2 × 10^4^ cells per well. Twenty-four hours later, diluted sterile milk samples, *β*-casomorphine-7, and *β*-casein were added to the cells. Cell viability was tested by using the Trypan Blue Dye Exclusion method. The percentage of viable cells was calculated according to the following obtained absorbance (ABS):

% cell viability=(ABS value of treated cells/ABS value of control cells) _x 100_.

### 2.5. Statistical Analysis

All data are presented as mean ± SD. Statistical analysis was performed using one-way analysis of variance ANOVA, SPSS version 20.0 (*p* < 0.05; statistically significant). *Post hoc* test was carried out to determine the differences: (a) between the means of treated groups and control group, (b) between the means of donkey colostrum and other groups, and (c) between the means of the groups of mature milk for each of 11 parameters (NF-*κ*B, Akt1/2/3, p-Akt1/2/3, Erk-1/2, p-Erk-1/2, JNK, p-JNK, p-STAT-1, and CD26).

## 3. Results

In this study, we investigated whether the exposure of skin fibroblasts to different milk species, *β*-casein, and *β*-casomorphine-7 affects cell viability, inflammatory response, and proliferative pathways.

### 3.1. MTT Assay

The MTT assay explored cellular metabolic activity and cell viability. Based on or experimental MTT assay data, none of the milk species and peptides cultured with skin fibroblasts exerted a significant influence on cell viability, except *β*-casomorphine-7.

### 3.2. Flow Cytometric Analyses

**Figure 1:**
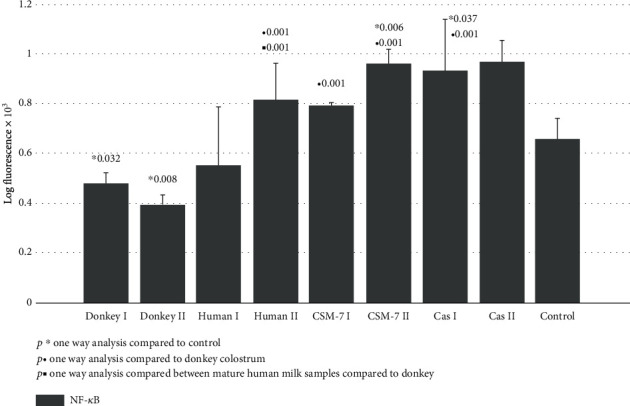
NF-*κ*B p65 signaling of skin fibroblast cultures exposed to different milk samples, *β*-casein, and *β*-casomorphine-7. Fibroblasts exposed to the following milk species: donkey colostrum (donkey 1); donkey mature milk (donkey 2); human colostrum (human 1); human mature milk (human 2); casein 1/10 (cas I); casein 1/100, (cas II); beta-casomorphine-7, 1/10, (CSM I); beta-casomorphine-7, 1/100 (CSM II); and control group. Each group comprised of 4-6 culture samples.

Significant downregulation of NF-*κ*B p65 subunit expression was detected in skin fibroblasts exposed to donkey milk samples (colostrum and mature milk), compared to the intact fibroblasts. On the other hand, its quantitative expression was especially pronounced in fibroblasts, exposed to *β*-casomorphine-7 and *β*-casein I (Figure 1).

**Figure 2:**
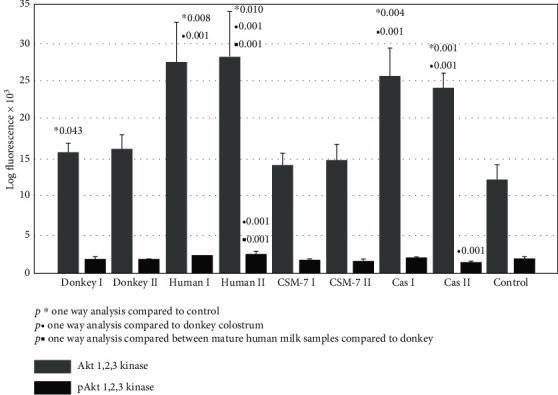
Akt1/2/3 kinase and p-Akt1/2/3 kinase signaling of skin fibroblast cultures exposed to different milk samples, *β*-casein, and *β*-casomorphine-7. Fibroblasts exposed to the following milk species: donkey colostrum (donkey 1); donkey mature milk (donkey 2); human colostrum (human 1); human mature milk (human 2); casein 1/10 (cas I); casein 1/100, (cas II); beta-casomorphine-7, 1/10, (CSM I); beta-casomorphine-7, 1/100 (CSM II); and control group. Each group comprised of 4-6 culture samples.

**Figure 3:**
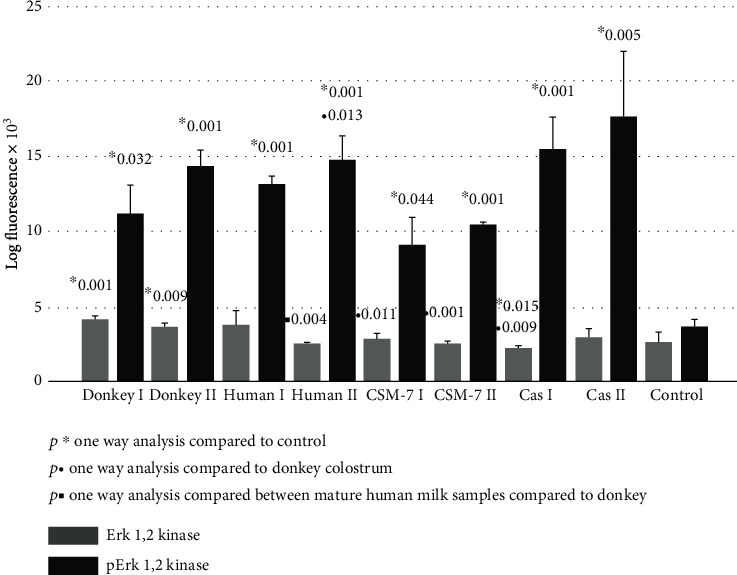
Erk1/2 kinase and p-Erk1/2 kinase signaling of skin fibroblast cultures exposed to different milk samples, *β*-casein, and *β*-casomorphine-7. Fibroblasts exposed to the following milk species: donkey colostrum (donkey 1); donkey mature milk (donkey 2); human colostrum (human 1); human mature milk (human 2); casein 1/10 (cas I); casein 1/100, (cas II); beta-casomorphine-7, 1/10, (CSM I); beta-casomorphine-7, 1/100 (CSM II); and control group. Each group comprised of 4-6 culture samples.

Significant upregulation of Akt1/2/3 kinase after fibroblasts exposure to the above mentioned milk species and *β*-casein was documented. The activity of its active phosphorylated form (p-Akt1/2/3) did not increase significantly (Figure 2). Contrary to Akt1/2/3 kinase, the Erk1/2 kinase active-phosphorylated form (p-Erk1/2) was upregulated almost three to four times in all treated groups. The most remarkable effect of about fivefold increase was observed after *β*-casein treatment (Figure 3).

**Figure 4:**
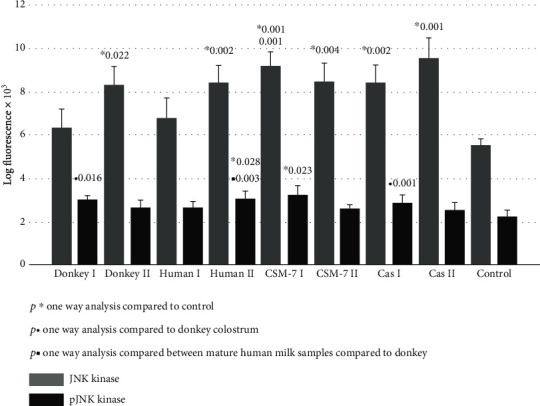
JNK kinase and p-JNK kinase signaling of skin fibroblast cultures exposed to different milk samples, *β*-casein, and *β*-casomorphine-7. Fibroblasts exposed to the following milk species: donkey colostrum (donkey 1); donkey mature milk (donkey 2); human colostrum (human 1); human mature milk (human 2); casein 1/10 (cas I); casein 1/100, (cas II); beta-casomorphine-7, 1/10, (CSM I); beta-casomorphine-7, 1/100 (CSM II); and control group. Each group comprised of 4-6 culture samples.

**Figure 5:**
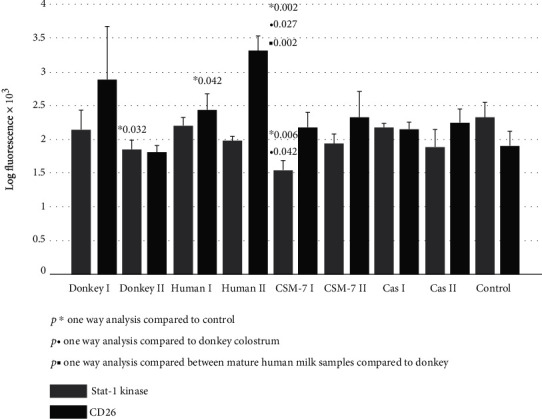
p-STAT-1 kinase and CD26 signaling of skin fibroblast cultures exposed to different milk samples, *β*-casein, and *β*-casomorphine-7. Fibroblasts exposed to the following milk species: donkey colostrum (donkey 1); donkey mature milk (donkey 2); human colostrum (human 1); human mature milk (human 2); casein 1/10 (cas I); casein 1/100, (cas II); beta-casomorphine-7, 1/10, (CSM I); beta-casomorphine-7, 1/100 (CSM II); and control group. Each group comprised of 4-6 culture samples.

Figure 4 represents the quantitative expression of proliferative downstream signaling pathway of the JNK kinase. All investigated treatments upregulated JNK kinase and p-JNK kinase up to 45%. A significant decrease in activated Stat-1 kinase (p-Stat-1) expression was documented following a fibroblasts' culture treatment with mature donkey and cow milk (Figure 5). The quantitative expression of surface antigen CD26 was significantly upregulated after treatment with human milk (Figure 5).

## 4. Discussion

The human skin is the largest organ of the body, continuously exposed to a variety of environmental influences. Such influences may induce skin aging, inflammation, or serious damage to the skin. The main challenges in treatment of chronic wounds are to stimulate skin regeneration, to decrease inflammation, and to preserve the skin smoothness and elasticity [4, 8–12]. Skin fibroblasts play a key role in the production of extracellular matrix, collagen, and elastin [4, 9]. Milk treatment can soothe irritated skin, can hydrate dry skin, and can replenish lost nutrients. It has been reported that skin wrinkles were treated with implanted autologous fibroblasts, which resulted in a significant improvement in periorbital skin flaccidity [33–35].

Donkey milk has been famous throughout history for its nutritional, therapeutic, and cosmetic properties [23, 24]. Both colostrum and milk from donkeys may be useful in the treatment of human immune-related diseases [26–28]. The low allergenicity of donkey's milk may be due to its low casein content, since the main allergens in cow's milk are caseins (*α*s1 and *β* type). The proportion of noncasein (whey) proteins is 35-50% in donkey milk, while in cow's milk these proteins represent about 20% [25, 30, 31]. Since fibroblasts may actively participate in inflammatory and immune responses, we evaluated the expression of the main redox-sensitive transcription factor activation, activator of inflammatory response, and the NF-*κ*B-p65 subunit. The expression of its active p65 subunit decreased significantly after donkey milk treatment but increased after the treatment with *β*-casein and *β*-casomorphine-7 (Figure 1). This suggests that noncasein bioactive peptides of donkey milk may be responsible for anti-inflammatory property of donkey milk and colostrum [36]. It has been reported that free radical activation of NF-*κ*B may occur under the stimulation of fibroblasts with cytokines (TNF-*α* and IL-1), bacterial TLR-4 activating product lipopolysaccharide (LPS), reactive oxygen species (ROS), and other DNA-damaging agents. Activated NF-*κ*B may induce the expression of inflammatory cytokines, matrix-metalloproteinases (MMP), and collagenases, responsible for ECM destruction [32, 33]. The overexpression of NF-*κ*B is involved in fibroblast apoptosis, together with the increased Bax ratio [33]. Our results suggest that donkey milk may ameliorate free-radicals induced inflammatory skin disorders through the NF-*κ*B active p65 unit suppression of the skin fibroblasts.

In our study, we further investigated a possible favorable effect of donkey milk, compared to casein and *β*-casomorphine-7 and to human milk species, on skin fibroblasts' proliferative potential. We demonstrated that among the kinase pathways, the most susceptible was Erk1/2 kinase (Figure 3), which was upregulated in all tested samples at least three times. Intracellular signaling pathways responsible for survival, proliferation, and differentiation consist of a family of different serine/threonine/tyrosine kinases, uniquely activated by dual phosphorylation of corresponding residues upon stimulation by growth factors, cytokines, hormones, or other mitogens. MAP kinases are grouped into three families, which are extracellular-signal-regulated kinases (Erks), Jun amino-terminal kinases (JNKs), and stress-activated protein kinases (p38/SAPKs) [15–17]. The activation of Erk kinase occurs by phosphorylation of Thr202/Tyr204 motifs. Once activated, p-Erk may activate about 150 downstream cell-signaling proteins, responsible for survival, cell cycle activation, proliferation, differentiation, and cell metabolism [17–19, 37]. The activation of Erk-1/2 is able to inhibit apoptosis induced by oxidative stress, osmotic stress, hypoxia, or by growth factor withdrawal. It occurs by external Fas death receptors and TNF-related apoptosis inducing ligand (TRAIL) [38]. In addition, Erk activity is reduced in the adult skin compared to young individuals [39]. All tested milk species and *β*-casein were able to potentiate p-Erk pathway, which may suggest that the essential amino acids of casein may be responsible for Erk-induced cell cycle activation, proliferation, differentiation, and cell metabolism. Akt kinase family includes serine/threonine kinases, which play a fundamental role in regulating cell metabolism, proliferation, and survival. Their continual activation may lead to the development of cancer phenotype, while Akt1/2 knockout mice, which have dwarfism and skin, muscular, and bone atrophy, die immediately after birth [38]. Serine/threonine phosphorylation is needed for full Akt kinase activation, but in our study, we detected only pAkt1/2/3 activation after exposure to human milk and *β*-casein (Figure 2). In addition, we found that increase of JNK and the p-JNK pathways is far less than that of p-Erk (Figure 4), indicating that stimulation is shifted in favor of the Erk activity. The phosphorylation of JNK at Thr183 and 185 positions is required for its full activation. It has been reported that JNK takes part in biosynthesis of ECM, remodeling of fibroblasts into myofibroblasts, and matrix contraction after stimulation by inflammatory cytokines and growth factors [40–42]. In addition to MAP kinases, several members of the signal transducers and activators (Stat), which belong to a family of transcription factors, especially Stat-1, may be recruited by growth hormone stimulation. The results of our study suggest that the Stat signaling pathway was suppressed after exposure to donkey milk and *β*-casomorphine-7 (Figure 5). Fibroblasts act as antigen-presenting cells in a number of immune and inflammatory reactions, by expressing various antigens on their surface, by secreting inflammatory cytokines (IL-1, IL-6, TNF-*α*, and GMCSF) and chemokines (IL-8, RANTES, eotaxin, and MCP). Surface antigen CD26, which exerts dipeptidyl peptidase-4 (DPP4) activity, is a typical marker for fibroblast lineage. Its importance in fibrosis and scar prevention was documented by using specific inhibitors against DPP-4 [8]. No significant increase in the CD26 expression was observed after the cells were exposed to donkey milk, what consistently correlates with an absence of inflammatory response in skin fibroblasts (Figure 5).

Possible limitations of the study lie in the fact that the metabolic properties of fibroblast cell culture may vary in early and late cell generations, showing *in vitro* senescence, ageing, and differentiation [43].

In conclusion, the downregulation of the redox-sensitive inflammatory transcription factor NF-*κ*B pathway observed after skin fibroblast exposure to donkey milk may suggest that noncasein bioactive peptides of donkey milk may be responsible for the anti-inflammatory properties. This may suggest usefulness in the treatment of inflammatory skin diseases. Another important finding of our study is that among cell signaling molecules, the most sensitive downstream effector is p-Erk kinase. Stimulated p-Erk pathway may point to donkey milk usefulness in wound healing, regenerative, and aesthetic dermatology.

## Data Availability

All data used were included within the article.
